# LOOKING FORWARD: BEHAVIORAL RISK FACTOR SURVEILLANCE SYSTEMS (BRFSS) CAREGIVING DATA

**DOI:** 10.1093/geroni/igz038.676

**Published:** 2019-11-08

**Authors:** Lisa C McGuire

**Affiliations:** CDC, Atlanta, Georgia, United States

## Abstract

The Centers for Disease Control and Prevention (CDC), through its Behavioral Risk Factor Surveillance System (BRFSS), collects data on caregivers and the caregiving situation as well as many health behaviors, annually. The BRFSS is the world’s largest ongoing health survey, administered in all 50 U.S. states, as well as the District of Columbia and the three U.S. territories, with data collected from more than 400,000 respondents. In 2015-2017, the 9-item caregiving module was administered in 44 states, DC, and Puerto Rico on the BRFSS. CDC’s Alzheimer’s Disease and Healthy Aging Program has developed many data for action resources for use by states and other partners to help identify populations and communities most at need. This presentation will describe future enhancements with the BRFSS caregiving module and the series CDC developed resources to facilitate date utilization, including state-specific infographics, data briefs, and the online data portal.

